# Moving targets: threat and uncertainty on the front line

**DOI:** 10.3389/fpsyg.2026.1888719

**Published:** 2026-08-04

**Authors:** Cade McCall, Aaron Laycock

**Affiliations:** Department of Psychology, University of York, York, United Kingdom

**Keywords:** cognitive performance, military psychology, stress, threat, training

## Abstract

**Introduction:**

Programs for training individuals to work in hazardous environments should be based on a clear understanding of how extreme danger affects cognitive performance. But our understanding of life-threatening experiences is necessarily limited by the fact that we cannot practically or ethically study them in the controlled context of the laboratory.

**Methods:**

Here we draw upon first-hand accounts of individuals who work under serious threat to explore potential gaps in the research on, and training for, cognition under threat. We conducted intensive one-on-one interviews with 10 members of highly specialized military forces, each of whom provided moment-to-moment accounts of life-threatening events. They also shared beliefs about optimal traits of team members and training approaches.

**Results:**

Thematic analyses revealed important insights about the variety of threatening experiences that military personnel confront. Key themes emerged regarding the inherent unpredictability of operational environments and the effects of that unpredictability on affect, cognition, and behaviour. Participants highlighted the necessity of cognitive flexibility when confronting ongoing changes in the environment. They also highlighted the unavoidable challenge of making critical decisions amid uncertainty. Critically, participants raised serious doubts about the ability of either training programs or intelligence gathering to adequately prepare personnel to confront unpredictability.

**Discussion:**

Based on these insights, we discuss the necessity of research and training to tackle the interactive effects of threat and uncertainty. We further discuss how nontraditional warfare and recent technological additions to battlespace (e.g., autonomous systems) amplify this necessity, and how innovations in training might address these challenges.

## Introduction

Surviving in a life-threatening environment requires more than brute strength and quick reflexes. It also depends upon complex cognitive responses such as assessing risk, making quick decisions, and improvising actions. How does the mind respond to these diverse and ever-changing demands while under extreme stress? What cognitive and emotional mechanisms allow some individuals to better thrive amidst hazards? And can these mechanisms be cultivated? These are important questions both for basic research on the nature of the human threat response and for applied research on performance in threatening environments.

Accordingly, empirical research has revealed a great deal about threat and performance over the past decades (Eysenck et al., 2007; Pessoa, 2009; Robinson et al., 2013; Tomaka et al., 1993; Vuilleumier, 2005). This research has examined the threat response at different levels, from neural activity (Adolphs, 2013; Tovote et al., 2015; Wilson-Mendenhall et al., 2013), to peripheral physiology (Tomaka et al., 1993), subjective experience (LeDoux and Hofmann, 2018; McCall et al., 2015) and behaviour (McCall et al., 2016; Roelofs et al., 2010). This large body of research reveals that threat impacts cognitive performance in a variety of different ways, including shaping attention (Eysenck et al., 2007), perception (Vagnoni et al., 2012), decision-making (Akinola and Mendes, 2012; Clark et al., 2012; Laycock et al., 2024), and memory (Palombo et al., 2015). Findings furthermore reveal important individual differences in individuals’ abilities to adaptively respond to threat (Hildebrandt et al., 2016; Seery, 2011; Waugh et al., 2008) in the moment. In the long term, exposure to threat is further associated with potential effects on cognition and mental health (Bogliacino et al., 2017; Gade and Wenger, 2011; Giannouli, 2024).

Nevertheless, capturing and characterising the response to serious threats is not straightforward. Laboratory-based research relies upon a variety of methods to emulate them using threat of pain via electric shocks (Cantelon et al., 2019; Clark et al., 2012; Grillon et al., 2004), social threats (Kirschbaum et al., 1993), threatening images (Balaban and Taussig, 1994), and threatening immersive virtual environments (McCall et al., 2015). But for obvious reasons, researchers cannot place human participants in actual danger. As a consequence, lab-based studies have a necessary limit that likely constrains our understanding of how the experience of threat evolves over time, as well as our ability to identify key individual differences that determine one’s capacity to face severe and prolonged periods of threat. These are serious shortcomings. Selection and effective training are essential to survival for individuals working in these environments, but identifying the right person for the job and knowing when they are ready is notoriously difficult (Cardona and Ritchie, 2007; Harden et al., 2021; Vanhove et al., 2016).

Given these constraints, first-hand accounts of individuals who have repeatedly confronted life-threatening danger can provide unique insight (Flood and Keegan, 2022). Indeed, interview-based techniques have recently been used to examine the effects of threat on memory (McKinnon et al., 2015; Palombo et al., 2015), decision-making (Shortland et al., 2020), and team performance (Dixon et al., 2017; Ramthun and Matkin, 2014). The current study takes a related approach to examine the time course of threat, examining ongoing changes in cognition, affect, and behaviour across accounts of threatening experiences. We furthermore explored the beliefs of individuals with extensive experience with mortal danger regarding the traits and training that best prepare individuals for handling these environments. Specifically, we conducted intensive and extended interviews with 10 individuals who had served in highly specialized military units. Each of these individuals had multiple, significant close combat experiences to draw upon. Furthermore, the interviews were conducted by a recently retired member of the military (the second author) who, as a consequence, shared some common ground from which to build rapport as a peer. Here we report key themes from these interviews and tie them back to contemporary issues in threat-related research and military training.

## Methods

### Participants

All 10 participants in this study served in highly specialized, highly trained military units. Participants were recruited via email or via opportunity sampling via social networks. All were men, ranging in age from 33 to 45 years (mean = 39). Each participant had extensive experience in the military (mean years in service = 16.5) and multiple operational tours in frontline combat roles. Participant ranks ranged from lance corporals to lieutenant colonels. Approval for recruitment and data collection was granted by the ethics committee of the Psychology Department at the University of York.

### Procedure

Each interview was conducted face-to-face by a retired member of the military. The interviews lasted between 40 and 75 min. Before beginning each interview, we explained the nature of the study and acquired informed consent. This included consent to record the interviews. We also asked participants to omit the names of individuals and the names of military operations when speaking about their experiences. When participants inadvertently disclosed any of this information, we immediately removed it from the audio recording.

The interviews were semi-structured in nature and consisted of two sets of questions. In the first set, we sought to gather detailed subjective accounts of experiences in threatening environments. These questions were based on phenomenological approaches (Giorgi, 1997; Hein and Austin, 2001; Klein and Westcott, 1994), as well as research on autobiographical memory (Levine et al., 2002). To elicit descriptions of a variety of experiences, we asked two questions. For Question 1, we asked participants to “briefly describe a direct action that went as you expected.” (“Direct action” is military parlance for a short duration military operation with an identified objective within a hostile or potentially hostile environment.) Once they had provided an overview of the event, we asked participants to “walk us through this event from start to finish. Take us step by step through your experience—all the details you remember about the scene, thoughts, and feelings.” When participants finished answering this question, the interviewer delivered follow-up probes (e.g., “Do you have any other memories of what you were thinking or feeling?”) The format of Question 2 was the same as Question 1, but this time we asked participants to, “briefly describe a direct action that *did not* go as you expected.” We varied the questions in this way in order to elicit a range of experiences that varied in their risks and demands. We also wanted to encourage participants to go beyond idealised or archetypical examples.

The second set of questions was designed to directly probe participants’ beliefs about optimal strategies for working under these threatening conditions, and whether or not those strategies could be cultivated through training. To elicit these ideas, we asked the following questions: (a) “Are there any mental states or mindsets you feel are best suited to operational sentry duty, patrol route out of FOB, or direct action?,” (b) “Do you think this was addressed in the way you have been trained to deal with hazardous environments?,” and (c) “If you were putting together a team of people who might confront either type of situation, what traits would you look for?”

### Analysis

Although we asked each participant the same set of questions, the interviews were nevertheless conversational in nature. As such, participants occasionally jumped between topics, going back to flesh out their answers to a previous question. With that in mind, we did not segment the content into the individual questions for analysis. Instead, we analysed the transcripts as a whole.

We used reflective thematic analysis (Braun and Clarke, 2006, 2022) to identify themes in the data. The analysis process was collaborative (involving the two authors) with intermittent discussions to settle on codes and themes, as described below (Patton, 2014; Richards and Hemphill, 2018). We took a descriptive approach focusing on the participants’ reported experiences. While our approach was primarily inductive, we were undoubtedly influenced by our own theoretical interests in the effects of threat and uncertainty on performance. Our analyses followed the phases put forth by Braun and Clarke (2006). In the first phase, we each familiarized ourselves with the data by both listening to, transcribing, and reading, each interview in its entirety. In the next phase we worked together to generate initial codes. Each rater then went through the interviews independently and applied codes to passages, adding and discussing novel codes when necessary. We pooled our respective sets of passages in one aggregated spreadsheet. Next, we collaboratively organized the codes into potential themes and then, independently, took turns going through the coded passages to review each theme’s content. Finally, we discussed and named each theme. We worked together to produce the report, i.e., the Results section, in which we summarized the themes, relying heavily on compelling extracts from the original interviews. We relate these themes to the broader literature in the Discussion section.

## Results

### Question set 1: experiences

When asked to walk us through past threatening experiences, participants provided a range of different examples, from planned direct actions to patrols to being ambushed. While the details of these experiences varied widely, a common distinction emerged between moments when a threat was more ambiguous or looming versus moments when the threat was obvious and concrete. Some accounts included both, with a clear turning point between these “left-of-bang” and “right-of-bang” moments. To capture these distinctions, we have divided this section into subsections describing left-of-bang versus right-of-bang moments. A third subsection summarizes participants’ descriptions of the immediate aftermath of threatening experiences, when the threat had subsided but continued to influence the mind and body.

#### Left-of-bang

Even during high conflict operations, direct encounters with the enemy are relatively rare and often brief. However, military personnel spend extended periods of time in left-of-bang situations, when hazards such as IEDs (improvised explosive devices), ambushes, or other forms of attack might be imminent.

Uncertainty pervaded accounts of these moments, ranging from acute uncertainty about the specific situation at hand to more general uncertainty about its ultimate outcome. According to participants, this uncertainty is an inevitable feature of military operations. Despite extensive training and planning, it emerges, e.g., “Even though we had rehearsed it…rehearsals are rehearsals, is not a live target. That was the bit of reality.” As another participant explained, experience is no cure, “[You] do not really know what is going to happen, even [with] previous experience. Nothing is set in stone. You think it’s going to go one way. It might potentially not, [so you are] going [into an] unknown situation really.”

For obvious reasons, these moments were also characterised by subjective experiences of foreboding. Participants described an “airy uncomfortable feeling” and an uneasy quiet (e.g., “I just remember this feeling amongst us, this quiet. Quietness.”). One participant provided a detailed account of waiting within enemy territory for orders to proceed,

“[It was] quite eerie…It’s not knowing what else is out there. Are you going to perform? Are you going to take a casualty? Are you going to be a casualty? …You are stuck behind the burn thinking, ‘I’ve got this’. But then there’s the unknown of, what is out there? What is the enemy? And who’s going to engage, and with what?”

In terms of cognitive responses to these contexts, the combination of uncertainty and looming threat facilitated heightened vigilance. Several participants mentioned being “switched on” for long periods in order to avoid IEDs. When in vulnerable locations, “We all had our head on a swivel” and “your senses would heighten a little.” One participant explained the link between emotion and this vigilance,

“There is definitely some fear. [It] felt like there was sort of heightened sense of awareness, because we knew we [were] going into their [territory]. Almost like a hyper-alertness, probably borne out of fear, like more than ‘I…want to do my job right’. Of course you do. But…everyone [had] heightened awareness. I think out of fear more than anything else.”

Further evidence for this heightened vigilance emerged in the remarkable detail of participants’ accounts of left-of-bang moments. These included descriptions of the weather (e.g., “a bright day, like not a cloud in the sky. Not particularly [hot] because the time of year…But, you know, a dry and hot day. Not like 50° hot, but…up to mid 20s, I guess.”) as well as of landscapes and cityscapes (e.g., “I remember how close and how built the area was. I remember…everything being on top of you.”).

Of course, the vigilance described in these moments served to anticipate danger. As one participant explained, his goal was to “pre-see” the enemy. Participants described being attuned to both minute and global changes in the scene. In terms of minute changes, one participant explained, “It was anticipating…thinking [about] every little movement in front. Could that be a firing point or anything like that?” In terms of monitoring global changes, two participants mentioned moments when they noticed a general change in the social environment; civilians began gradually clearing out from an area which suggested that an enemy attack might be coming (e.g., “I was thinking, we have gone from pretty normal…almost a bit boring, to… ‘Something is going to happen here.’”)

As these descriptions suggest, a great deal of thought during left-of-bang moments was dedicated to imagining both unseen and future events. Participants constructed mental models of what was happening across a given operation, “I just remember looking at the compound for hours and hours and hours wondering exactly what was going on…behind the scenes…Because obviously there’s a lot going on, and you just do not get to hear about it.” Along similar lines, participants reported mentalizing about the thoughts and plans of enemy combatants, “You know you have landed. You know people know you have landed. You cannot avoid that.”

These mental models, in turn, facilitated the construction of different action plans. One participant described his thoughts while on patrol in a potentially hostile area,

“The first part [of my thoughts] would be an IED blast. In the second part would be like a scenario. If somebody was to shoot me what would I do here? And about backup. Try [to] play-set things for your mind: where the better cover is, always plays [a] scenario in your head.”

Naturally, left-of-bang threats wax and wane over the course of a military operation. Participants mentioned that over the course of a deployment, this ever-changing sense of looming danger had a cumulative effect on stress.

#### Right-of-bang

Accounts of right-of-bang moments were markedly distinct from left-of-bang moments and the transition between the two was abrupt. One salient feature of this shift from unspecified threat (e.g., the danger of being shot at) to concrete threat (e.g., being shot at) was physiological,

“There was an adrenaline rush … a quick reaction, whenever we received a bit of incoming… whether you expect it or not, it still puts the [hair on the] back of your head up.”

For some participants, this physiological change scaled directly with the concrete nature of the threat; the more concrete the threat, the stronger the response (e.g., “I did not really kick until shots fired, when we had [seen] the weapons”). As one participant described it,

“My heart rate went off when I realised I’d been quite close [to incoming bullets] … I do not think my reaction was as strong as it would have been if I’d seen the shooter, because it was just a random [shot]. I could hear the gunfire and I could hear it was a bullet when it went past. But it’s always a lot more [when] … you can see the enemy. You’ve always had a far greater response, your heart rate has gone up a lot more, had a greater impact on you then just coming under small arms fire from somewhere.”

Participants further explained that these intense moments of “adrenaline through the roof” are not sustainable for long periods. Describing an event in which he was attacked repeatedly, one participant explained, “The first time I was on a high that carried on to the second time but … I do not know if you have only got a limited amount of adrenaline over a certain period… [but by the] third time, I was just like…‘Get out of here.’”

Besides the surge of energy, participants did not tend to recall feeling much emotion right-of-bang. As one participant said, “At the time, I did not actually feel too much because [it] happened so quick between…weapons seen, shots fired.” When participants did describe emotional qualities of right-of-bang experiences, they were not necessarily negative. Participants described experiencing more excitement than fear, perhaps because “you are actually doing the job that you want to do…” This sense of excitement seemed to change over the years for some individuals,

“Over my career, my feelings of fear have changed…[During] my first experience, I do remember …excitement, and it’s funny how you can conflict between fear and excitement really quick and if you have got fearful excitement, it’s got more energy…it sort of feels like it courses through your veins. If you have not had a cigarette for a long time, and then you have one, it’s the same feeling. But as time has gone on, stepping away from the [battle] scenario, you get fearful, particularly since having been married and had children. You get more fearful for those whose safety’s resting in your hands.”

Despite what some of these quotes might suggest, none of the participants expressed pleasure in violence itself. Instead, right-of-bang moments represented an opportunity to move from a state of apprehension to taking clear and decisive action. As with the physiological response, participants frequently described this shift into action as instantaneous, “I was patrolling along the river, we got engaged from bushes at the other side, literally dropped straight into the ditch, and was returning fire without, it was just instant.”

Some participants described this shift into action as “training mode” or the “training taking over.” One participant explained, “You do not really think about much because the training takes over. So it’s just like you are buzzing because this is something you are trained for and it’s actually happening.” Although these quotes about a training mode might suggest automaticity of behaviour, right-of-bang accounts were not characterised by the mindless execution of a preplanned set of actions. Participants described intense cognitive activity, although it was distinct from left-of-bang moments in the sense that attention was narrowly focused and geared toward action. One participant described the response to being attacked as “tunnel vision.” Another participant described being under heavy fire, “I did not focus on anything else, except what we were doing. I did not have time to have a pregnant pause and reflect on all the cool stuff I’ve done in my life. I just did not think about it.” This intense focus apparently afforded clarity, “So, I think all the contacts I was involved in, it was surprising the clarity that you found descended upon you…I can still vividly remember it.”

One apparent by-product of the intense focus of right-of-bang cognition is that peripheral events or stimuli that would normally be salient are not so, or are even ignored completely. In one example, a participant described being shot but not noticing they had been hit or feeling pain until long after the right-of-bang moment. Additional evidence for this extreme focus emerged from participants’ descriptions of time passing during right-of-bang moments. As one explained, “I do not know how long that went on for and I cannot remember now…it went in a split second but it must have been hours by the time we finished.”

Regardless, some stimuli seemed to break through the intense task focus during right-of-bang moments. For several participants, these were particularly brutal images of physical injury and death. Several participants mentioned clear memories of seeing or smelling blood. As one participant explained, “I remember smelling a lot of blood,…But [I] do not think that anything [else] really stood out.”

As these accounts suggest, it was not merely the perception of blood and injury, but empathic responses appeared to penetrate task focus, e.g., “I do remember how pale the casualties were, you know they [were] in a lot of pain.” One participant explained that this is particularly powerful when assisting injured civilians,

“I had a woman that was screaming and shouting at me… in [another language], which I did not understand. I had the smell of this burnt skin, in my arms, of a kid…I remember the mother… she starts screaming, lowering the cloth around this baby. Yes it fucking knocks you back a bit. You almost become numb to what you see every day. If you have adults with blood and guts hanging out, it’s not really bother you. Just crack on. And then you get given that…It’s hard to try and focus on the job. I was more bowled over by the mum, thinking, ‘shit, just look at her, she has had her life destroyed.’ There is a lot of things going through your mind at that point…. Feelings wise, I think it was more…toward the mother, the gran, That is grim…It’s a bit of realism then, because it’s not a bloke, it’s not mates, it’s a kid. That’s tough.”

#### Release from threat

Although we did not ask participants to describe the moments after their threatening experiences, they tended to do so spontaneously. Naturally, these moments (i.e., the moments after a right-of-bang encounter) had their own distinct qualities in participants’ memories. Participants reported an amping down of the physiological response. “You can breathe again” and can take “a deep breath and a sigh.” These physical states were not altogether pleasant. Participants reported feeling exhaustion and even waves of nausea. As one participant explained, “[I] start feeling a bit sick. I feel the adrenaline come down…I was shaking, like tingling, tingling fingers, probably the effect of the adrenaline, slowly coming back down.” For at least one participant, the release from immediate threat was accompanied by the emergence of intense physical pain from injuries incurred in the preceding event.

Not surprisingly, participants reported experiencing a profound sense of relief after the immediate threat had passed. But the emotional experience during these periods varied dramatically and could be quite mixed. On the one hand, participants described celebratory moods, particularly with regards to group dynamics. As one participant recounted, “At first it [was] all laughing…. We absolutely smashed it. Did it correctly. Everything is fine.” But the release from threat also had challenging aspects. As one participant explained, “it’s just a completely different shift [of] emotion in your brain…once it’s over, I just remember that sense of emptiness. I just felt hollow inside….[I was] on my own, thinking, ‘What …just happened’?.” This sense of astonishment was shared by several participants (“that actually happened”; “you sit down, and you go, ‘…what happened today?’”).

**Figure 1:**
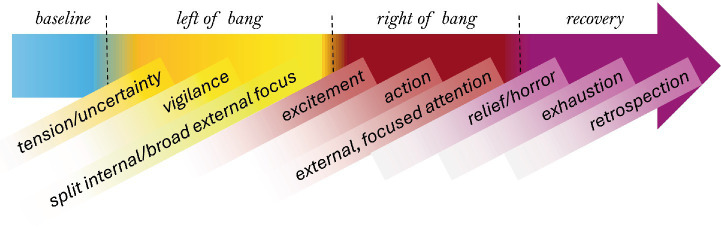
Depiction of the three experience-related themes.

As these quotes indicate, participants’ thoughts quickly shifted to retrospection--trying to make sense of the threatening experience itself. One participant explained, “Once it got quiet and … there was a plan in place, the heart rate goes down and you start thinking about things more logically.” Participants went over the events, “Did everything go right? Could you done anything differently?” These inner monologues seemed geared toward evaluating one’s performance and considering alternative outcomes, e.g., “It did not go as planned. [It] went as well as it could …. I would not have done anything different, had I known the way it was going to go.” Participants furthermore seemed to weigh the experience and its relevance for their futures, “It’s done now. That was the first one on the way. I can do this. It’s fine.” These included thoughts about how things might have turned out otherwise, e.g., “And I remember thinking, that was … close. If we had been a split-second earlier… We were just lucky that nobody got hit” (Figure 1).

As that last quote demonstrates, this period of reflection was potentially disturbing in and of itself. Moreover, the impact of this reflection period itself is potentially long-lasting. One participant explained, “Now if they had to send me to war, I would go, ‘I am aware that I’m not immortal now’.” Another participant explained that, for him, the period after a right-of-bang experience was the most disturbing part of the process,

“It wasn’t till afterwards that I was…really scared…Out of all those three phases--the before, during, and after--it was the after for me. The after is what I think about a lot. Rarely do I ever think about, weirdly, what happened and the time before it. I always [harken] back to how I felt when I was on my own [in hospital].”

### Question set 2: beliefs

The second set of questions in our interviews probed participants’ explicit beliefs about the types of traits or “mindsets” that are optimal or suboptimal for military work in threatening environments. We divided this section into individual theme-based subsections. The final subsection goes beyond descriptions of specific traits or mindsets and delves into participant ideas about optimal and suboptimal approaches to training.

#### Flapping

When asked who they would put in their ideal work team, participants had a nearly universal response: “no flappers.” Here flapping refers to a state when an individual responds to a threatening situation by being incapable of thinking or acting meaningfully. Not surprisingly, flapping was both loathed and feared; flappers were perceived as dangerous colleagues and even more dangerous leaders.

Participants explained flapping as a kind of mental freezing. As one participant described it, “moving, but I froze in [the] mind.” Examples of flapping included behaving frantically (“throw everything up in the air,” “running in circles”) or irrationally. One participant elaborated on the subjective feeling of flapping,

“The physiological side, for me, takes over your mental side because your mental side is trying to focus on how to get … out of here. But then [there is a] sick feeling, [a] hollow feeling. You are like, ‘Oh this is not good.’ …. You got lost in that world of… hopelessness.”

Several participants shared theories for the underlying causes of flapping. These theories attributed flapping to the shock and confusion caused by confronting something completely unexpected or unfamiliar, e.g., “Flapping, I suppose, is just you being unable to … compute what it is you are seeing or what you are being exposed to…” Flappers therefor fall short of “problem-solving. They cannot compute.” In this sense, flapping is not only about being frightened or overwhelmed with fear but about grappling with the unknown. As one participant explained in detail,

“I do not think it’s…fear as in scared. It’s fear as in unknown. Everything up until that point they have seen, they have been told about it… Like in my first tour, I froze, right at the end of the tour. I remember seeing two guys that had…drove over [an] anti-tank mine…[and had sustained serious injuries] I’ve never seen anything like that. It was so vividly raw. And there was running. I was moving, but I froze in my mind. I always think back to that… It’s you subconsciously checking out of the situation because you have seen something that is unknown. It’s uncharted territory, not necessarily scary, just really weird…. It wasn’t that fear of them dying. That emotion came afterwards. But it was just the fear of, ‘That was a really weird, that does not [seem]real.’ It was crazy.”

While participants’ most dramatic examples of flapping emerged during intense right-of-bang moments, participants also described similar performance issues during left-of-bang moments. Here, flapping took the form of acting prematurely when problem-solving and caution were necessary. One participant described younger soldiers who seemed to act compulsively under pressure, eager to drive through an area littered with IEDs, “that was the biggest thing with it. Lads: ‘Fuck it am driving straight through it.’ You are like, ‘No…Stop the wagon. No. Here’s a map. It’s got seven explosions at the same point. Why are you going to bother driving through it?… Where is your mindset going now? Because it takes 2 minutes to stop get out check it, what’s the point of risking it?’…”.

Crucially, nearly all of the participants explained that one cannot predict how a person will perform under extreme stress until they encounter it themselves, e.g., “It does not matter how strong you are, you do not know how you are going to react. Everybody can gob off as much as they want. When it actually happens, you do not know how you [are] going to … react.” The unpredictable nature of operational experiences seems to contribute to the unpredictability of one’s response. As one participant explained, “when it happens, they have no build up to it, no conditioning to it. We do not have [control over] when that’s going to happen, how it’s going to happen, and who it’s going to happen to. So, I think all of those factors [are] built-in as well, as to the strength or the impact, of the shock, what causes that freeze.”

#### Calm

Given the risks of flapping, it is not surprising that almost all of the participants highlighted the need for individuals to be emotionally stable and relatively calm under pressure. They wanted “cool heads, no flappers,” “calm people that were not really excitable…[without] a reputation for overreacting, making a drama,” “somebody [who] did not get stressed out [easily].” Participants acknowledged that operational environments are inevitably stressful, but explained that soldiers then need to be “comfortable with the uncomfortable.” Several participants further explained that being calm is not simply a chronic state but an ability to regulate one’s emotions. As one participant explained, “People do get stressed out. It’s just a natural daily thing. But somebody that is working themselves up and cannot settle themselves” will have a problem in these settings.

#### Vigilance

One of the key benefits of remaining calm, according to several participants, was the ability to stay vigilant and attuned to the ongoing changes in the environment, e.g., “you got to have that composure to read the environment, read where you are. That’s critical.” “Information and situational awareness is really important.” As one participant explained, “Calm is being able to process what your senses are telling you, what your senses [are] seeing or reading or feeling.” Individuals need “to be able to take themselves out of that [panicked] state” to be “aware of the wider environment and engage the controllable.”

The ability to maintain vigilance is also important in the face of potential boredom and distraction. Soldiers need “a high tolerance of repetition to get good”; “you have got to deal with boredom.” One participant highlighted the fact that distraction and boredom can be difficult to spot within a team, “a preoccupied guy is never good. I mean it’s difficult to say you cannot be preoccupied. Some people are preoccupied and they will never tell you. But you do need to be totally focused on what you are doing and not having your mind wander anywhere else.”

#### Behavioural flexibility

Several participants explained that the unpredictability of operational environments requires individuals to adjust quickly in order to take action when action is needed, e.g., “Do not do nothing, do something. So, I do not want a [person] that’s just going to freeze…it’s the last thing you want.” One participant described the ideal team member as, “quite cool, calm, collected, but at the same time somebody that…can get really aggressive at the flick of a switch.” Conversely, participants highlighted the need to be able to turn off aggression as soon as it is unnecessary,

“You cannot have some sort of Viking raid where everybody is going berserk, because you very quickly end up breaking the law of armed conflict and exacerbating the very situation of why those people are fighting against you in the first place…You need somebody who … is fairly calculating, who can turn that dial to go extreme aggression and then turning off as soon as the proportion of violence diminishes through its requirements and its delivery.”

#### Cognitive flexibility

Participants also discussed the challenges of problem-solving and decision-making under threat. They explained that optimal performers need to be “analytical” and “deep thinkers” with “a good balance of academic knowledge and common sense” who are “naturally inquisitive” and “critical thinkers.”

Participants repeatedly discussed the fact that the unpredictability of the operational environment calls upon a unique set of cognitive skills that may have otherwise not be obvious, e.g.,

“I have worked with people before who are great when things go down the rail tracks that they perceive this day is going to go. These rail tracks [go] straight down the line and they are absolutely great in those situations. But then the as soon as somebody changes the tracks and your train goes off a different path or different route they are fucked.”

Accordingly, optimal performance requires some degree of improvisation and “thinking outside the box.” As one participant said, “[There’s] no black-and-white option in this. It’s always the least worst option.” Another participant elaborated,

“You have to be able to think on your feet. [It’s] great being able to work out the biggest sum in your head, but that’s not going to help you think on the ground. There are ways of doing things better, more efficiently and quickly.”

Participants also explained that optimal performance relies upon taking in a great deal of information about the context and making quick sense of it. One participant explained that,

“some people…get to information overload a bit quicker than others… They tend to be the flappers from my experience. The people that can spin four or five plates and know what is going on… do not tend to flap as much… There is loads of information.”

One key cognitive challenge, then, is absorbing and prioritising information. Because the number of variables might be overwhelming, one “need[s] to be clever in the ability to assimilate a lot of information and prioritise.” Because the best action is not always obvious, one can only “focus on what I do know.” One participant summarized the challenge, “It requires the level of intellect to do all those really difficult things in a situation where you probably end up knowing as much as you can see or hear in front of you.”

#### Independent thinking

Several participants emphasized the importance of independent thinking within teams. They wanted colleagues with the confidence, “to make the decision, ‘Yes, I’ve done it this way. But I’ve done it because of this …reason and it worked because I knew [it] was going to work.”

Participants believed that independent thinking was particularly important for groups to be successful, e.g.,

“You also want folk that [are] questioning enough to not allow you to get into groupthink and everybody starts nodding round the table at a fairly ropey plan…You do need a broad team, to make sure you are capturing all opinions as you go forward.”

Along those lines, participants wanted a colleague who “pipes up when he needs to pipe up, say something very constructive—“constructive criticism with a valid point.” Speaking up is also necessary, even if one does not have a concrete suggestion per se, “If something does not look right, we need to have the confidence to say, ‘I’m not sure,’ Which may make something else happen.”

Obviously, independent thinking raises the potential for conflict within a team. Accordingly, one participant explained that constructively communicating is key,

“You will say something and I might categorically disagree with it. And actually it might upset me as well, because it might be something close to my heart. The emotionally intelligent or critical thinker [says], ‘Well, I appreciate what Aaron is saying actually, and he has got these experiences, and looking at it from this point of view, hence why he thinks that. But he might not have this experience that I have, and hence why I feel so strongly about it.’ So I need to check myself as well. That’s what I really look for in guys. I think it’s really important, especially now. Operating environments, whether we [are] going against a state actor or a nonstate actor…the days of just doing a section attack… are long gone.”

#### Prosociality

Along similar lines, participants mentioned a variety of prosocial traits that they wanted in team members. Participants repeatedly mentioned that they needed to be able to trust the members of their team. They wanted teammates who were “unselfish,” “open and honest with everybody,” people who “want to fight for me, I want to fight for them because they are my mates.” They wanted a “very conscientious, not showie type of person, that has a lot of considered thought.” Other participants also wanted teammates with “humility” and “reliability.” These traits were considered important given the team-based structure of the work, “yes there is a leader, but the responsibilities are shared equally… the burden is distributed more equally amongst the team and sometimes selfish and stubborn people aren’t good at that.”

Another desirable social factor that emerged in several interviews was a sense of humour. One participant explained, “To me the key is and always will be the black humour. The black humour is the bit that gets you through whatever is. So no matter how bad it’s been, at the end of it you can have a laugh and a joke and talk about it.” Another participant explained that a sense of humour is important for the group as a whole because,

“Without a sense of humour…it can put a right downer the situation. That can affect the whole troop…Negativity is toxic…When you can laugh it off, albeit you do not particularly want to, it sometimes does help. Being able to perk people up, take your head out what you are thinking about, totally refocus people…Before you know it they have forgotten about why they was feeling bad…just a little diversion.”

#### Self-awareness

Another trait that came up in several interviews was self-awareness. Participants pointed out the critical role that self-awareness plays in learning from stressful situations when performance was suboptimal. One participant explained, “I met people as well that…do not recognise when they are flapping. Because I have been in situations where I have flapped, but then [I know] I was flapping there. I wasn’t performing optimally because I have let the flapping takeover…I can recognise when I have a flap and recognise it had been detrimental.” This recognition then facilitates learning from the event. Along these lines, one participant described an event early in his career when he had flapped. Although this event was only during a training exercise, it shaped his subsequent approach to work and remains a source of motivation,

“It’s fear of failure that drives me. I use that rather than when people do visualisation, ‘Oh do you remember that time when I smashed that?’…you do not do that as a [member of this unit]. You hark back to, ‘I do not want to be that bloke.’ So I’m going to put more effort on this detail, another two hours on this report, get someone else to check it. I’m going to go the extra distance, so I do not get that sensation again. It’s that fear of that. It do not affect everybody. You talk to a lot of sports people, they talk about visualisation of the euphoric thing of winning, stuff like that. For me it’s not like that. I do not really get off on that feeling, I’d rather not have the bad feeling, than the good feeling.”

#### Training

Over the course of the interviews, participants discussed the degree to which training prepares individuals for operational environments. Participants consistently mentioned that their specialized unit’s training regimen prepared them for the intense physical demands of ground operations, e.g., “You come out [of training with] the mindset [that] you know your body can practically do anything. If you put your mind to it, your body will follow.” Participants further explained that the drilling of specific tasks within military training (e.g., break contact drills) automatized those behaviours such that one could execute them under extreme pressure, e.g., “Because of muscle memory you know exactly what is supposed to do and without really having to think about it.”

But participants also explained that training did not necessarily prepare them for the psychological strain of these situations. Some participants were sceptical that training could ever prepare an individual for “the shock…Even though training is as good as it can be…I do not think you can ever mitigate that and I do not think necessarily everybody can react in the same way.” Other participants raised similar points regarding the distinct nature of direct experience, e.g., “Unless you have experienced it, [it] is quite hard to describe. Nobody else can experience someone else’s emotion.” Perhaps for this reason, several participants felt that direct experience, and only direct experience, best identifies people who can perform optimally (e.g., “experience outranks the rank”) and uniquely prepares individuals for future encounters with danger (e.g., “once you start surviving those sorts of things you become comfortable with the uncomfortable”).

While these quotes suggest that training might never match the benefits of direct experience, participants nevertheless suggested ways to prepare individuals for working in threatening environments. As one participant argued, “Everybody can be coached to process things more calmly, react differently, in stressful situations. We have to expose people to stressful situations for them to do that. I do not think anybody is a natural at … reacting in pressure situations. I think everybody’s trained.”

So, in lieu of direct experience, what are the best training practices? According to several participants, accurately simulating operational environments is critical, e.g., “If you put a bloke in [a] scenario over and over again, sooner enough he will stop flapping about that scenario because he knows you got to do it. Then when it comes to happen in real life, he does not think…He just does it.” Participants discussed simulating shocking or disturbing combat experiences via exposure to the flesh and blood injuries, e.g., “live flesh training, stuff like that, just seeing blood…I think [that is the only way] to build that threshold,…or some way of simulating it.” One participant also discussed the method of “having actual amputees coming and [simulate] those wounds. I think that would have gone a long way to helping, because subconsciously you would hark back to the training, ‘Right…I’ve seen this.’…I think that’s the only way to get round it. That’s what I would have liked to have had, to prepare me.” This idea was echoed by another participant who was in favour of training courses that “have lads that are amputees…play the amputee on the ground and you have to administrate him.”

Critically, participants suggested that the cognitive side of these tasks should simulate the unexpected or unpredictable challenges of actual operations (e.g., because “lads flap about that, because it is the unknown. And they do not know what to expect…what’s going to come.”).

Participants contrasted these more naturalistic means of simulating cognitive and emotional stressors with some of the more traditional means employed within the military, such as sleep deprivation or trainer evaluation, e.g.,

“You can put somebody under pressure by saying, ‘This is a test. You are not going to have any sleep. You are going to have somebody of a high rank breathing down your neck.’ That is pressure. But it’s not the pressure of, ‘Go and clear that room.’ You clear that room and you actually get shot by simulated round and you get the fucking pain of it, almost like the embarrassment of getting shot, but you can actually go back and be like ‘Fucking hell, I cannot enter a room like that again. Otherwise I would get shot for real.’ Whereas blank rounds and you are tired—that pressure is not as realistic as it could be…Because there are two different types of pressure. I think people shouting at you and keeping you awake for ages is pressure. But I feel like being shot with simunition… would be more realistic…When you want to train somebody to be skilful, doing it when somebody has not slept for three days—[they] are not going to be performing optimally. They’re not going to perfect that skill.”

Participants also argued that the constant assessment within the training context can backfire. Trainees can end up focusing on impressing the trainer and not practicing the critical skills, e.g., “Nobody wants to be a failure. Nobody wants to be looked at as weak. You’re a [a member of an elite regiment]. Not one [member] has ever been weak and they think, ‘I do not want to be that first ever one to turn around and say no I cannot do that, cannot do this.’” One of the key problems with the evaluate-and-punish approach, according to several participants, is that it hampers learning. Trainees end up, “freezing from not wanting to get something wrong…They do not want to go outside the boundary, go outside the box to get it done. That freezes them.”

Instead, several participants suggested, trainees should have the opportunity to fail. Accordingly, they supported more recent developments in training strategies that use failures as a learning tool, e.g., “Go, ‘Right—we failed. How do we correct that failure instead of trying to mask it?’ We will succeed…If you only succeed, [then] when you [are] put on your arse, you are not going to know how to get up.” In terms of a learning process, participants described learning from failure as a coaching process, e.g., “If someone is not dealing with it too well, you can try and train and talk through what he has done and over a period of time you need to make sure he is getting that.”

Finally, participants also suggested that failure or flapping within the training environment can provide an opportunity to cultivate resilience to the stress of working under threat. As one participant explained,

“It might be his first exposure to it, so he might never have seen this before. It’s all new to him. [He] does not know how to react to it. His mates to his left and right might have seen all this before. It’s not their first operational tour maybe. That’s where you have got to let the blokes bring him on, ‘Listen I remember the first time I did it and I flapped,’ and stuff like that. ‘Look, now I deal with the situation. I have gone through the training you have gone through. You will be sound,” and talking him through it. ‘Do you need any tuition?’ Try and bring him on that way.”

## Discussion

The participants in this study shared richly detailed accounts of their experiences in threatening environments. They also shared their ideas about who excels in these environments and the traits and training practices that might enable them to do so. The themes that emerged point to key questions for basic research and highlight important issues for screening and training of combat personnel and other high-risk occupations.

At a basic level, the participants’ accounts illustrate the multidimensional and dynamic nature of threatening experiences; threats and their psychological responses evolve over time. This fact emerged most clearly in the contrast between left-of-bang and right-of-bang moments, when possible threats become concrete. Left-of-bang moments were characterised by apprehension, vigilance, distributed attention, and the precautionary generation of hypothetical scenarios, while right-of-bang moments were characterised by intense physiological arousal, sharply focused attention on the scene, and action. Participants’ accounts of operational exposure further highlighted the significance of the period immediately after a threatening experience. Remarkably, multiple participants described these post-threat moments as overwhelming in and of themselves as cognition shifted into reflection on the threatening experience, their actions within it, and its actual or alternative outcomes.

These qualitative changes over the time course of threat are obviously driven by the changing demands of the situation. Nevertheless, the distinctions are worth noting. Right-of-bang situations (e.g., being under direct fire) might provide an archetypical example of threat and might, as such, drive our ideas about who can handle threatening environments and how they can do so. But soldiers spend a great deal more time in left-of-bang situations (e.g., on high-risk patrols). These periods may take a unique kind of toll in terms of stress and fatigue. Although they may be more nebulous in nature, the choices made during left-of-bang moments can nevertheless have serious, even lethal, consequences. Along the same lines, the intense reflective period immediately after a threat subsides may seem inconsequential but it is likely to play a critical role for cognition and learning and, in turn, for determining future performance under threat.

Of course, performance was a key concern for all the participants. Failure at a critical moment (i.e., “flapping”) was perceived as both humiliating and dangerous. Narratives of flapping varied dramatically from moments in which an individual froze while unable to comprehend their surroundings, to moments when people launched into irrational action that did not address the problem at hand. Regardless, flapping seemed to emerge when one’s psychological response was at odds with the current state of the environment, perhaps responding with a left-of-bang mindset to a right-of-bang moment or vice versa (i.e., thinking when action was needed or acting when thinking was needed).

With all this in mind, it is perhaps not surprising that participants highlighted the importance of both calm and flexibility in the field. Their ideal team members can adjust to the changing situation, flip into or out of action, and maintain self-control throughout. They have the vigilance required left-of-bang and the action readiness required right-of-bang. In this sense, participants sought team members whose responses were as dynamic as the environment itself.

In terms of the broader literature on threat, the current work speaks directly to recent calls from prominent researchers to take subjective experience seriously (LeDoux and Hofmann, 2018; McCall et al., 2015). More specifically, these interviews suggest that when basic research accounts for the full time course of threatening experiences (Davis et al., 2010; Kuppens et al., 2010; Lehman et al., 2015) and when it examines qualitative changes across physiology, affect, cognition, and behaviour (Hildebrandt et al., 2016; Tomaka et al., 1993), it will be the most fruitful in understanding performance under threat. Indeed, the distinction between left-of-bang and right-of-bang experiences described here maps nicely onto research in psychology and neuroscience on the diverse nature of threat responses. Specifically, the notion of the threat continuum (Fanselow, 2018) describes functionally distinct states that allow an individual to respond in a way that befits the proximity and certainty of the threat. Recent work further links these different defensive states to distinct neural networks and responses (Mobbs et al., 2020). Seen through this lens, the participants’ reports of flapping may be instances of a mismatch between an individual’s neural response within the threat continuum and the actual demands of the environment.

Along these lines, the findings speak to the need for cognitive, affective, and behavioural flexibility when confronting the dynamic nature of threatening environments (Harris et al., 2017; Oden et al., 2015; Pulakos et al., 2002). Our interviewees repeatedly highlighted the pervasive role of uncertainty in threatening environments. Left-of-bang experiences were characterised by the unpredictable or ambiguous nature of the threat itself, while unfamiliar scenarios caused flapping or freezing during right-of-bang experiences. Even after threats had subsided, participants sought certainty regarding their actions and the outcome of the event. Similarly, participants identified the ability to cope with uncertainty as an optimal trait or mindset for working in threatening environments. They highlighted the need for individuals who can handle deviations from a plan and can choose the “least worst option” when given incomplete or confusing information. Participants further highlighted the need for independent thinking and constructive communication to facilitate group decision-making under uncertainty.

All these points suggest that training should prepare individuals for the potent combination of threat and uncertainty. Of course, doing so is a significant challenge. Traditional approaches to training, particularly in the military, necessarily include drilling of required skills and may involve rehearsing decision-making frameworks (Shortland et al., 2020). But drilling and scripted rehearsal in highly controlled training environments might not prepare one for the inherent unpredictability and dynamism of real-world threats (Delahaij et al., 2006; Van Den Heuvel et al., 2012). Instead, training approaches that deliberately introduce uncertainty (Simpkin and Schwartzstein, 2016) and require learning from errors (Eva, 2009) may prove beneficial. More broadly, basic research on threat and uncertainty (Laycock et al., 2024; Neta et al., 2017; Power and Alison, 2019; Schmitz and Grillon, 2012) should be brought to bear when considering strategies for both screening and training for individuals who cope well with uncertainty under different types of pressures.

Technologies such as virtual reality might be particularly helpful for training and testing complex thinking under threat. The military sector has long been at the forefront of extended reality (XR) development (Pallavicini et al., 2016). Standard XR training simulations may be missing a trick by providing tools that only allow users to rehearse a fixed set of actions. XR simulations are easily changeable, so each run through a simulation is potentially unpredictable. Introducing more of this intrinsic uncertainty to training simulations may help train and test the ability to cope with uncertainty in real time. Indeed, XR-based research has demonstrated that threat can affect decision-making under uncertainty (Laycock et al., 2024). Training should leverage XR’s ability to produce complex, not just photorealistic, simulations.

Along the same lines, XR-based training can explore the whole continuum of threat. Much of the focus of XR-based training simulations has been on the fidelity of environments and interfaces. But XR provides additional capabilities in amplifying emotional experience. XR is a powerful tool for presenting acute threats that elicit right-of-bang states (Hildebrandt et al., 2016), as well for creating ambiguous or looming threats that elicit left-of-bang states (McCall et al., 2022). As research cited above suggests, the mind, brain and body responds differently to these different environments (Mobbs et al., 2020). At the very least, training should tap this whole range to best prepare personnel for the breadth of possible operational experiences.

The individuals interviewed for this study served in an era when autonomous systems were only beginning to emerge as a common feature of warfare. The introduction of these technologies, as well as the broader use of AI in battlespace, raises new concerns regarding the effects of threat and uncertainty on human operational performance. Most obviously, the use of autonomous systems by adversaries makes battlespace more complex. As evidence from Ukrainian soldiers demonstrates (Cox, 2025), the presence of drones requires a constant level of left-of-bang style vigilance that is both cognitively and emotionally taxing. More broadly, the war in Ukraine and the propaganda campaigns around it have demonstrated the power of AI-produced media to seed confusion in militaries as well as the broader population (Paziuk et al., 2025).

Autonomous technologies do not only amplify threat and uncertainty because they are in the hands of adversaries. They also introduce new complexities to users and operators. As these interviews made clear, no amount of training or intelligence can prevent uncertainty in operational environments. Amid this inherent confusion, AI-based systems may offer a false sense of certainty. Human monitors are a standard requirement for military autonomous systems because no AI system is perfect; training data and faulty assumptions can compromise the most sophisticated systems (Probasco et al., 2025). But operational environments put human monitors under extreme stress. And research suggests that both threat and uncertainty may affect automation bias (i.e., the bias to delegate control to automated systems;) (Rosbach et al., 2025; Zhang et al., 2024). As such, the introduction of autonomous technologies should be tightly coupled with both Human-Robot and Human-AI interaction research (Adams et al., 2024; Kox et al., 2022; Probasco et al., 2025).

The themes in these interviews are the insights of a very specific population; all the participants in this study are male members of highly trained and highly specialized military forces with direct experience of combat. Indeed, we chose this sample because of their experiences in life-threatening environments. The fact that the main interviewer has a similar background also likely helped encourage rapport and the ability to use a common language (Bonner and Tolhurst, 2002). Nevertheless, any specific sample will provide sample-specific insights. Our interviewees were recruited to, and chose, a career in potentially hazardous environments. A more complete picture should include individuals from other communities who deal with life threatening environments, e.g., first responders (Cogan et al., 2024) or from individuals exposed to threatening environments outside of the professional realm, e.g., survivors of natural disasters or random acts of violence (Bista and Sharma, 2019). A broader picture would also benefit from voices that differ in terms of gender and culture, particularly given the fact that identity can shape the ways in which individuals describe, and are affected by, threatening experiences (Giannouli, 2024).

Regardless, the insights of the participants in this study illustrate that performance in life-threatening environments requires not just strength, but also cognitive, affective, and behavioural flexibility. Research and training should seek to cultivate that flexibility in military personnel, particularly as new forms of autonomy increase the complexity of battlespace.

## Data Availability

The datasets presented in this article are not readily available because they are interview transcripts that include personal information about the participants. The original transcripts are not available for sharing. Inquiries regarding the datasets should be directed to Cade McCall (cade.mccall@york.ac.uk).
